# Catalogue of herpetological specimens of the Ewha Womans University Natural History Museum (EWNHM), Republic of Korea

**DOI:** 10.3897/zookeys.965.52976

**Published:** 2020-09-03

**Authors:** Yucheol Shin, Yikweon Jang, Steven J. R. Allain, Amaël Borzée

**Affiliations:** 1 Laboratory of Animal Behaviour and Conservation, College of Biology and the Environment, Nanjing Forestry University, Nanjing 210037, China Nanjing Forestry University Nanjing China; 2 Division of EcoScience and Department of Life Sciences, Ewha Womans University, Seoul 03760, South Korea Ewha Womans University Seoul South Korea; 3 Department of Biological Sciences, College of Natural Science, Kangwon National University, Chuncheon 24341, South Korea Kangwon National University Chuncheon South Korea; 4 Durrell Institute of Conservation and Ecology, School of Anthropology and Conservation, University of Kent, Canterbury, United Kingdom University of Kent Canterbury United Kingdom

**Keywords:** Amphibia, herpetology, historical collection, museum specimens, Reptilia, Republic of Korea

## Abstract

The herpetology collection of the Ewha Womans University Natural History Museum (EWNHM) represents one of the oldest and largest institutional collections in the Republic of Korea. The specimens deposited in the EWNHM represent a major historical collection of the native herpetofauna, both in species diversity and time span. However, the full inventory of the herpetology collection has never been conducted and thus the collection has received little attention from researchers. Here, the first full account of the herpetology specimens held at the EWNHM is provided, with voucher information for all documented specimens to make the collection accessible for future studies.

## Introduction

Natural history collections are an invaluable repository for modern biological research. These collections have broad applications including the detection of faunal changes, species decline, biogeography, systematics, and species discovery (Shaffer et al. 1998; Graham et al. 2004; Wójcik et al. 2010; Lister et al. 2011). A series of specimens collected over time also serves to shed light on changes in genetic diversity and morphology that occurred during that time period (Babin-Fenske et al. 2008; Cornetti et al. 2018). In order to conduct such collection based research, a full inventory of specimens held at the museum is a valuable resource as it saves time locating specimens, designing studies and managing collections.

In the Republic of Korea, early efforts to collect herpetological specimens were conducted by foreign researchers. Thus, vouchered specimens collected during this time were deposited in natural history museums outside of the country (American Museum of Natural History 2020; e.g., a series of *Bombina orientalis* vouchers collected in early 1950s). Although such specimens are a valuable historical collection, they usually do not fully encompass the native herpetofauna in taxa, time span, and geographic locations (American Museum of Natural History 2020; e.g., herpetological specimens historically collected in Korea). Moreover, natural history collections within the country are poorly known to researchers outside the institution where the collection is located and thus received little attention in herpetological research.

Ewha Womans University Natural History Museum (EWNHM; Sabaj 2016) was established in 1969 as the first natural history museum for the country (EWNHM website 2014). Although small in size, the herpetology collection of EWNHM is of great historical value for herpetological research, with some specimens dating back to early 1950s being collected during the Korean War.

Despite this great value, a complete catalogue of the collection has not been available. Although a public database (Korean Natural History Research Information System; NARIS) provides information on EWNHM specimens, this database is only a partial representation of the collection. Also, this database uses a different cataloging system from EWNHM, leading to voucher inconsistencies and potential problems of locating specimens. Therefore, a full herpetological inventory of the collection at EWNHM following a consistent format is needed for future utilization of specimens. Here, we provide the first complete catalogue of the herpetological collection of EWNHM, using consistent voucher system throughout specimens. In doing so, we also changed degrading labels to prevent the loss of important information and updated the nomenclature of species if the taxon underwent taxonomic revisions between the time of initial labelling and our cataloging effort. Also, we applied a new and consistent voucher system throughout specimens to resolve confusion of conflicting voucher systems. Although this means yet another change of voucher system applied to specimens, this catalogue can serve as a reference point towards reducing confusions originating from multiple conflicting voucher systems.

## Materials and methods

### Description of the collection

The herpetological specimens of the museum EWNHM are located in the wet collection room. The herpetology collection was roughly divided into reptiles and amphibians. For some species of anurans, 50 to 100 individual specimens were contained per glass jar (e.g., *Bombina orientalis*, *Glandirana emeljanovi*, *Pelophylax nigromaculatus*), with specimens packaged individually in small plastic bags. These specimens were mostly collected in one location on the same date, comprising voucher series of specimens. In some cases, specimens of one anuran species collected from two different locations were held in the same glass jar. In this case, specimens from two locations were divided into two by separate plastic bags. Some amphibian species were held in smaller glass jars with fewer number of individual specimens per container (< 25 individual specimens). We also found jars that contained two or more amphibian species collected from the same location. In this case, we either separated the species into separate jars or separated them into small plastic bags containing paper labels with the appropriate species information.

Specimens of lizards were housed in small individual containers or packed into small number of specimens (< 20 individuals per container) representing voucher series. Specimens of snakes and turtles were individually contained in glass jars. All specimens were preserved in 1% formalin solution at the beginning of collection and preservation process (Jaewon Ryu, pers. comm.). The use of preservative solution has not been changed over the years.

### General protocol for processing specimens

We used a table to mount a tripod and camera (Nikon D50; Nikon Corporation, Tokyo, Japan) in order to photograph the specimens. The table was covered with white synthetic fabric to provide a white background for photography. A 15-centimeter ruler was taped above the fabric to be used as a scale of reference. We used a whole-face respirator (3M Company, Minnesota, USA) to prevent potential toxic inhalation of preservative fluids. We also used industrial-grade wiper (Yuhan-Kimberly, Ltd, Seoul, Korea) to remove fluid spillage. For photography, we mounted the camera on a tripod with the lens facing vertically downwards. Each specimen was photographed in dorsal, lateral and ventral angles. Specimens of eggs and amphibian larva were not photographed to prevent potential damage caused by the handling process.

### Labelling protocol

As most of the Korean herpetofauna have undergone significant nomenclatural changes over the past 50 years, and as some of the labels showed signs of degradation, it was necessary to update the labels. In doing so, we kept the original labels alongside the new labels with updated nomenclature.

For labeling, we first prepared a general label for each specimen or series of specimens. This label contained key information about the specimen(s), including scientific name, Korean common name, geographic location of collection, collection date, and collector(s). The label was printed on regular A4 papers and was 8.8 × 5.7 cm in size. All relevant information were written on the labels using a pencil. The new labels were fully immersed in preservative fluid following previous collection maintenance practice of EWNHM.

Alongside the general label, it was necessary to assign an unique voucher number to each specimen that did not currently have one, according to the nomenclature rules of the museum. The preexisting voucher system for the museum collection is such as EWNM-AR-XXXX, “XXXX” being the serial number. However, this previous system was not consistently applied throughout all of the specimens held in the collection and needed to be corrected. Moreover, the voucher system represented in NARIS is different from the preexisting voucher system of EWNHM. Thus, in order to apply a consistent voucher number system to each and all herpetological specimens, the decision was made by the museum to discard both previous EWNHM and NARIS voucher systems and to use a new protocol for the herpetology collection. The new numbering protocol is comprised of museum code EWNHM, followed by a taxon code ANIMAL, and a four-digit serial number starting from EWNHM-ANIMAL 5279. Despite the change of protocol, the previous EWNM-AR voucher labels were retained alongside the new EWNHM-ANIMAL voucher labels for the traceability of information.

### Georeferencing protocol

One of the main issues concerning the use of natural history collections is determining the area of collection with reasonable accuracy (Newbold 2010). The collection labels, especially of historical collections, usually contain text-only geographic information of different extent and accuracy (Garcia-Milagros and Funk 2010). This was also the case with specimens deposited in EWNHM. Therefore, each specimen was georeferenced using the collection information available, with the locality of collection being recorded as latitude and longitude (in decimal degrees) describing the midpoint. For localities within a named town or city, the center of the settlement or named district was used as the midpoint. Google Maps was used along with the ‘What’s here?’ tool to display the latitude and longitude once the midpoint had been found. For those specimens collected in more rural localities, the midpoint of the named location was also used as in the urban ones.

### Catalogue

We organized the catalogue in Order – Family – Species order. For each species, English and Korean common names are also given alongside the current scientific name. The nomenclature used in the original labels is also given. Location and collector information (written in either Korean or Chinese characters) are directly translated.

### Terms and abbreviations used in the catalogue

**Leg.** Collector

**Figure 1.**
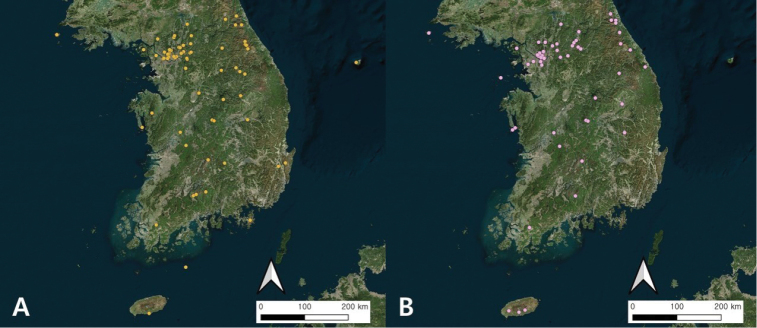
Collection localities of amphibian (**A**) and reptile (**B**) specimens of the Ewha Womans University Natural History Museum (EWNHM). Dubious collection localities are not shown in the map. Map generated in QGIS 3.10.0. The Bing Aerial Map was used as the base map, acquired via OpenLayers plugin implemented in QGIS 3.10.0.

**Loc.** Collection locality and collection date (see Table 1 and Table 2 for georeferenced GPS coordinates, and Fig. 1 for locations on the map)

**Juv.** Juvenile

**Nn.** Neonate

**Lar.** Larvae

**Td.** Tadpole(s)

**Egg.**Egg(s)

**Voucher series** A series of specimens of one species collected in the same location on the same date

-**do** Korean equivalent of province

-**ri** Korean equivalent of county

-**myeon** Korean equivalent of village

**Table 1.** Georeferenced collection localities of amphibian specimens deposited in the Ewha Womans University Natural History Museum (EWNHM). WGS 84 coordinate system.

Location	Latitude (°N) / Longitude (°E)
Baekryeongdo	37.9593N, 124.6654E
Baekwoondae	37.6598N, 126.9776E
Buhwangsa	37.6423N, 126.9713E
Buyong-ri, Yangsu-myeon, Yangpyeong, Gyeonggi-do	37.5551N, 127.3470E
Cheoneunsa, Jirisan	35.2728N, 127.4760E
Cheoneunsa, Jirisan	35.2728N, 127.4760E
Cheonmasan, Gyeonggi-do	37.6806N, 127.2727E
Chiaksan, Gangwon-do	37.3720N, 128.0504E
Jinburyeong	38.2652N, 128.3592E
Daeamsan, Inje, Gangwon-do	38.2118N, 128.1352E
Daedunsan, North Jeolla	36.1206N, 127.3232E
Dobongsan	37.7008N, 127.0156E
Dongducheon	37.9146N, 127.0746E
Dukcheon, Geomundo	34.0488N, 127.3184E
Gajwa-ri, Mitan-myeon, Pyeongchang	37.3109N, 128.5342E
Ganghwa	37.7134N, 126.4512E
Gapyeong, Gyeonggi-do	37.8115N, 127.4201E
Gayang Apt., Seoul	37.5639N, 126.8544E
Gayasan	35.8228N, 128.1179E
Geojedo	34.8542N, 128.6435E
Geomundo lighthouse	34.0491N, 127.3179E
Gimpo	37.6174N, 126.7088E
Godongsan, Gyeonggi-do	37.6586N, 127.4063E
Guri, Gyeonggi-do	37.5985N, 127.1282E
Gwangju, Gyeonggi-do	37.4061N, 127.3171E
Gwangneung	37.7522N, 127.1771E
Gwangneungnae	37.7462N, 127.2040E
Gyeryongsan	36.3427N, 127.2056E
Hongcheon, Gangwon-do	37.7390N, 128.0667E
Hwangyongdong 25-1, Gyeongju	35.8265N, 129.3691E
Hwayasan, Gapyeong, Gyeonggi-do	37.6716N, 127.4278E
Imjingak, Paju, Gyeonggi-do	37.8895N, 126.7401E
Jangsudae, Seoraksan	38.1196N, 128.3415E
Jeotgae, Anmyeondo	36.4229N, 126.4205E
Jingwansa	37.6381N, 126.9466E
Jirisan	35.3373N, 127.7305E
Jugeumsan, Namyangju, Gyeonggi-do	37.7845N, 127.2690E
Maesol Forest, Andong	36.5520N, 128.5901E
Mountain cabin of Chinbu-ryong, Gangwon-do	38.2652N, 128.3592E
Mugeuk, Eumseong	36.9960N, 127.5889E
Mujugucheondong	35.8775N, 127.7788E
Myeonggae-ri, Naemyeon, Hongcheon	37.8465N, 128.5373E
Myeongjisan	37.9420N, 127.4319E
Myeonmokdong, Seoul	37.5795N, 127.0972E
Namyangju, Gyeonggi-do	37.6563N, 127.2347E
Nanjido, Seoul	37.5715N, 126.8686E
Noewoon-ri, Pyeongchang, Gangwon-do	37.4142N, 128.3511E
Nogodan, Jirisan	35.2942N, 127.5326E
Noron-ri, Pyeongchang	37.3516N, 128.4317E
Odaesan	37.7986N, 128.5429E
Outside Jahamun, Seoul	37.5925N, 126.9664E
Pyeongnae, Gyeonggi-do	37.6524N, 127.2253E
Sanghwanam, Sokrisan	36.5469N, 127.8599E
Sangwonsa, Odaesan	37.7865N, 128.5643E
Seogwipo, Jeju	33.2495N, 126.5641E
Seoraksan Checkpoint	38.1200N, 128.4659E
Sinchon	37.5671N, 126.9432E
Sinwondong, Dukyang-gu, Goyang-si, Gyeonggi-do	37.6725N, 126.8791E
Sobaeksan	36.9521N, 128.4460E
Sokrisan	36.5336N, 127.8998E
Songchu Valley	37.7125N, 126.9849E
Soyosan, Dongducheon	37.9428N, 127.0878E
Sudeoksa, South Chungcheong	36.6630N, 126.6225E
Taehadong, Ulleungdo	37.5026N, 130.8247E
Under Ewha Bridge	37.6015N, 127.0724E
Wolchulsan, Yeongam	34.7746N, 126.7104E
Woljeongsa, Odaesan	37.7270N, 128.5956E
Woraksan, North Chungcheong	36.8894N, 128.0909E
Yangsuri, Gyeonggi-do	37.5702N, 127.3395E
Yongjang-ri, Namsan, Gyeongju, North Gyeongsang	35.7682N, 129.2254E
Yongwha-ri, Cheorwon-gun, Gangwon-do	38.1316N, 127.3480E

**Table 2.** Georeferenced collection localities of reptile specimens deposited in the Ewha Womans University Natural History Museum (EWNHM). WGS 84 coordinate system.

Location	Latitude / Longitude
Anmyeondo	36.4229N, 126.4205E
Baekdamsa, Seoraksan	38.1647N, 128.3738E
Baekryeongdo	37.9593N, 124.6654E
Balang-ri, Paju, Gyeonggi-do	37.8118N, 126.8969E
Biseondae, Seoraksan	38.1636N, 128.4658E
Bucheon, Gyeonggi-do	37.5013N, 126.7903E
Bukhansan	37.6611N, 126.9932E
Byeoksoryeong, Jirisan	35.3005N, 127.6446E
Cheonmasan	37.6806N, 127.2727E
Daedunsan, North Jeolla	36.1206N, 127.3232E
Daegwanryeong	37.6875N, 128.7604E
Deokjeokdo	37.2381N, 126.1279E
Ewha Womans University Campus	37.5598N, 126.9454E
Gajamul, Susaek, Seoul	37.5861N, 126.8952E
Gajeong-ri, Nammyeon, Chuncheon-si, Gangwon-do	37.7539N, 127.5829E
Gangchon, Chunseong-gun (=Chuncheon), Gangwon-do	37.8029N, 127.6138E
Ganghwa	37.7134N, 126.4512E
Godaedo	36.3892N, 126.3655E
Gomgol, Seoraksan	38.1200N, 128.4659E
Gotan, Chunseong-gun (= Chuncheon), Gangwon-do	37.9812N, 127.7149E
Gupabal	37.6365N, 126.9191E
Gwanaksan, Gyeonggi-do	37.4431N, 126.9610E
Gwangneung	37.7522N, 127.1771E
Gwangpan-ri, Chunseong-gun (= Chuncheon), Gangwon-do	37.7339N, 127.7014E
Gyeryongsan, South Chungcheong	36.3427N, 127.2056E
Hanlim, Jeju-do	33.3723N, 126.2912E
Hwajeon, Gyeonggi-do	37.6070N, 126.8738E
Jinburyeong	38.2652N, 128.3592E
Juan	37.4558N, 126.6828E
Jugeumsan, Gyeonggi-do	37.7845N, 127.2690E
Miro, Samcheok, Gangwon-do	37.4137N, 129.0621E
Mitan, Pyeongchang, Gangwon-do	37.3109N, 128.5342E
Mujugucheondong	35.8775N, 127.7788E
Myeongseongsan, Pocheon, Gyeonggi-do	38.1012N, 127.3493E
Outside Jahamun	37.5925N, 126.9664E
Palbongsan, Hongcheon, Gangwon-do	37.7032N, 127.7538E
Samakmyeon, Chunseong-gun (= Chuncheon), Gangwon-do	37.8401N, 127.6603E
Sangdodong, Seoul	37.4988N, 126.9382E
Sanghwanam, Sokrisan	36.5469N, 127.8599E
Sangwonsa, Odaesan	37.7865N, 128.5643E
Seongpanak, Jeju	33.3851N, 126.6204E
Seorim, Yangyang, Gangwondo	37.9680N, 128.5435E
Sinchon	37.5671N, 126.9432E
Sindangdong, Seoul	37.5523N, 127.0093E
Sindang-ri, Chungju	36.9101N, 128.0492E
Sokrisan, North Chungcheong	36.5336N, 127.8998E
Wolchulsan, Yeongam	34.7746N, 126.7104E
Yangju	37.8096N, 127.0305E
Yangsuri	37.5702N, 127.3395E
Yeongju	36.8115N, 128.5941E
Yeongsil, Hanrasan, Jeju	33.3489N, 126.4969E
Yukgokcheon, Euiseong	36.3459N, 128.6438E
Yumyeongsan, Gapyeong, Gyeonggi-do	37.5927N, 127.4911E

## Systematic account of vouchered specimens

### Class Amphibia Linnaeus, 1758

#### Order Anura Duméril, 1805

##### Family Bombinatoridae Gray, 1825

***Bombina orientalis* (Boulenger, 1890)**

Oriental fire-bellied toad; 무당개구리; 440 specimens.

Boulenger GA (1890) A list of the reptiles and batrachians of Amoorland. Annals and Magazine of Natural History, Series 6, 5: 137–144.

EWNHM-ANIMAL 5284; Loc: Dobongsan, 10 May. 1959; Leg: no data. EWNHM-ANIMAL 5286; Loc: Mountain cabin of Chinbu-ryong (= Jinburyeong), Kangwon-do, 24 Sep. 1977; Leg: Natural History Museum. EWNHM-ANIMAL 5292; Loc: Cheoneun-sa, 7 May. 1977; Leg: Yun Seokjun. EWNHM-ANIMAL 5293; Loc: Chinbu-ryong (= Jinburyeong), 12 Aug. 1980; Leg: Yun Seokjun. EWNHM-ANIMAL 5324; Loc: no data; Leg: no data. Voucher series EWNHM-ANIMAL 5325 – EWNHM-ANIMAL 5328 (total four specimens); Loc: Godongsan, Gyeonggi-do; Leg: Yun Seokjun. Voucher series EWNHM-ANIMAL 5329 – EWNHM-ANIMAL 5332 (total four specimens); Loc: Gapyeong, Gyeonggi-do, 5 Jun. 1982; Leg: Noh Bunjo. EWNHM-ANIMAL 5348; Loc: Daedunsan, Jeonbuk, 3 May. 1978; Leg: Natural History Museum. Voucher series EWNHM-ANIMAL 5349 – EWNHM-ANIMAL 5351 (total three specimens); Loc: Hwayasan, Gyeonggi-do, 20 Apr. 1997; Leg: Yun Seokjun. Voucher series EWNHM-ANIMAL 5364 – EWNHM-ANIMAL 5367 (total four specimens); Loc: Cheonmasan, 12 May. 1968; Leg: Noh Bunjo. Voucher series EWNHM-ANIMAL 5368 – EWNHM-ANIMAL 5371 (total four specimens); Loc: Dobongsan, 16 May. 1959; Leg: Kim and Lee. Voucher series EWNHM-ANIMAL 5410 – EWNHM-ANIMAL 5441 (total 32 specimens); Loc: Chinbu-ryong (= Jinburyeong), 12 Aug. 1979; Leg: Noh Bunjo. Voucher series EWNHM-ANIMAL 5442 – EWNHM-ANIMAL 5456 (total 15 specimens); Loc: Hwayasan, Gapyeong, Gyeonggi-do, 22 Apr. 1985; Leg: Yun Seokjun. Voucher series EWNHM-ANIMAL 5457 – EWNHM-ANIMAL 5486 (total 30 specimens); Loc: Chinbu-ryong (= Jinburyeong), 12 Jul. 1980; Leg: Yun Seokjun. Voucher series EWNHM-ANIMAL 5487 – EWNHM-ANIMAL 5514 (total 28 specimens); Loc: Sokrisan, 17 Jul. 1961; Leg: no data. Voucher series EWNHM-ANIMAL 5561 – EWNHM-ANIMAL 5660 (total 100 specimens); Loc: Gyeryongsan, 24 Jul. 1973; Leg: “Premed collecting team”. Voucher series EWNHM-ANIMAL 5661 – EWNHM-ANIMAL 5710 (total 50 specimens); Loc: Gyeryongsan, 23 Jul. 1973; Leg: Department of Biology. Voucher series EWNHM-ANIMAL 5711 – EWNHM-ANIMAL 5760 (total 50 specimens); Loc: Soyosan, Dongducheon, 11 Jun. 1972; Leg: Department of Biology. Voucher series EWNHM-ANIMAL 6060 – EWNHM-ANIMAL 6072 (total 13 specimens); Loc: Chiaksan, Gangwon, 2 Jun. 1979; Leg: Yun Seokjun. Voucher series EWNHM-ANIMAL 6073 – EWNHM-ANIMAL 6090 (total 18 specimens); Loc: Myeongjisan, 6 May. 1972; Leg: Jang Soonran. Voucher series EWNHM-ANIMAL 6091 – EWNHM-ANIMAL 6093 (total three specimens); Loc: Hwangyongdong 25-1, Gyeongju, 26 Apr. 2007; Leg: Yun Seokjun. Voucher series EWNHM-ANIMAL 6094 – EWNHM-ANIMAL 6110 (total 17 specimens); Loc: Cheonmasan, 19 May. 1979; Leg: Yun Seokjun. Voucher series EWNHM-ANIMAL 6111 – EWNHM-ANIMAL 6124 (total 14 specimens); Loc: Godongsan, Gyeonggi-do, 27 May. 1978; Leg: Yun Seokjun. Voucher series EWNHM-ANIMAL 6125 – EWNHM-ANIMAL 6128 (total four specimens); Loc: Buhwangsa, 3 May. 1964; Leg: Oh Soonja. Voucher series EWNHM-ANIMAL 6129 – EWNHM-ANIMAL 6143 (total 15 specimens); Loc: Dobongsan, Seoul, 16 May. 1959; Leg: Kim Myungae, Kim Myungsook, Lee Jongwan. EWNHM-ANIMAL 6144; Loc: Gwangneung, 11 May. 1957; Leg: Kang Jeon Il. EWNHM-ANIMAL 6355; Loc: Myeongjisan, Gyeonggi, 30 Sep. 2000; Leg: Yun Seokjun. Voucher series EWNHM-ANIMAL 6356 – EWNHM-ANIMAL 6358 (total three specimens); Loc: Chinbu-ryong (= Jinburyeong), 13 Aug. 1979; Leg: Noh Bunjo. EWNHM-ANIMAL 6362; Loc: Outside Jahamun, Seoul; Leg: Noh Bunjo, Yu Seongin. EWNHM-ANIMAL 6366; Loc: Gwangneung, 11 May. 1951; Leg: Kang Jeon Il. EWNHM-ANIMAL 6367; Loc: Woraksan, Chungbuk, 20 Jul. 1972; Leg: Noh Bunjo. Voucher series EWNHM-ANIMAL 6372 – EWNHM-ANIMAL 6373 (total two specimens); Loc: Jangsudae, Seoraksan, 10 Oct. 1970; Leg: “2^nd^ grade students”. EWNHM-ANIMAL 6378; Loc: Mujugucheondong, 9 May. 1979; Leg: Yun Seokjun. Voucher series EWNHM-ANIMAL 6391 – EWNHM-ANIMAL 6396 (total six specimens); Loc: Maebong, Odaesan, 6 Aug. 2001; Leg: Kim Byungwoo. Voucher series EWNHM-ANIMAL 6397 – EWNHM-ANIMAL 6400 (total four specimens); Loc: Gayasan, 17 May. 1963; Leg: Noh Bunjo. EWNHM-ANIMAL 6634; Loc: Woljeongsa, Odaesan, 16 Sep. 2006; Leg: Kim Byungwoo. EWNHM-ANIMAL 6661; Loc: Maebong, Odaesan, 8 Aug. 2006; Leg: no data. Voucher series EWNHM-ANIMAL 6672 – EWNHM-ANIMAL 6674 (total three specimens); Loc: Daeamsan, Inje, Gangwon-do, 18 May. 1993; Leg: Sung Gisoo.

**Citation**: Shin et al. (2020a).

**Remarks**: vouchers EWNHM-ANIMAL 5437, 5449, 5463, 5624, 5642, 5663, and 5677 had malformed limbs (Shin et al. 2020a)

##### Family Bufonidae Gray, 1825

***Bufo gargarizans* Cantor, 1842**

Asiatic toad; 두꺼비; 28 specimens

Cantor T (1842) General features of Chusan, with remarks on the flora and fauna of that island. Annals and Magazine of Natural History, Series 1, 9: 481–493.

EWNHM-ANIMAL 5279; Loc: Sudeoksa, 8 Jun. 1969; Leg: Eom Jeonghui, Lee Hyeonju, Lee Okju, Choi Jeongran. EWNHM-ANIMAL 5280; Loc: Jeongneung, Gyeonggi, 8 Jun. 1967; Leg: Paik Hyangsun. EWNHM-ANIMAL 5281; Loc: Gwangneung, Gyeonggi, 17 May. 1964; Leg: Lim Jeonghye. EWNHM-ANIMAL 5282; Loc: no data; Leg: no data. EWNHM-ANIMAL 5288; Loc: Muju Gucheondong, Jul. 1972; Leg: Yun Seokjun. EWNHM-ANIMAL 5289; Loc: Chinbu-ryong (= Jinburyeong), 13 Aug. 1980; Leg: Yun Seokjun. EWNHM-ANIMAL 5290; Loc: Eoreumgol, Cheonwang-sa, Kyungpook, 23 Jul. 1986; Leg: Yun Seokjun. EWNHM-ANIMAL 5291; Loc: Sinchon, 21 Jun. 1955; Leg: Department of Biology. EWNHM-ANIMAL 5294; Loc: Dongducheon, 1 Jul. 1971; Leg: Lee Eunbok Voucher series EWNHM-ANIMAL 5299 – EWNHM-ANIMAL 5301 (total three specimens); Loc: no data; Leg: no data. Voucher series EWNHM-ANIMAL 5302 – EWNHM-ANIMAL 5303 (total two specimens); Loc: no location data, 8 Aug, 1983; Leg: no data. Voucher series EWNHM-ANIMAL 5304 – EWNHM-ANIMAL 5305 (total two specimens); Loc: Mugeuk, Eumseong, Chungbuk, 31 Mar. 1994; Leg: Seo Hyeongseok. Voucher series EWNHM-ANIMAL 5306 – EWNHM-ANIMAL 5307 (total two specimens); Loc: no data; Leg: Kim Hungyu. EWNHM-ANIMAL 5308; Loc: Sanghwanam, Sokrisan, 17 Jul. 1961; Leg: no data. Voucher series EWNHM-ANIMAL 5309 – EWNHM-ANIMAL 5310 (total two specimens); Loc: Sinchon (Seoul), 5 Jul. 1955; Leg: Department of Biology. Voucher series EWNHM-ANIMAL 5311 – EWNHM-ANIMAL 5316 (total six specimens); Loc: Geojedo, 23 Jul. 1970; Leg: Kim Hungyu. EWNHM-ANIMAL 6724 (Egg.); Loc: Mugeuk, Eunseong, 31 Mar. 1994; Leg: Seo Hyeongseok.

***Bufo stejnegeri* Schmidt, 1931**

Korean water toad; 물두꺼비; 27 specimens

Schmidt KP (1931) A new toad from Korea. Copeia 1931: 93–94.

EWNHM-ANIMAL 5285; Loc: no data; Leg: no data. Voucher series EWNHM-ANIMAL 5811 – EWNHM-ANIMAL 5816 (total six specimens); Loc: Myeonggye-ri (= Myeonggae-ri), Naemyeon, Hongcheon, 21 May. 1992; Leg: Yun Seokjun. Voucher series EWNHM-ANIMAL 5817 – EWNHM-ANIMAL 5826 (total ten specimens); Loc: Seoraksan Checkpoint, 12 Oct. 1977; Leg: Donation by Prof. Yun Ilbyung. Voucher series EWNHM-ANIMAL 6369 – EWNHM-ANIMAL 6371 (total three specimens); Loc: Jangsudae, Seoraksan, 10 Oct. 1970; Leg: “2^nd^ grade students”. Voucher series EWNHM-ANIMAL 6627 – EWNHM-ANIMAL 6631 (Juv.; total five specimens); Loc: Maebong, Odaesan, 6 Sep. 2006; Leg: Kim Byungwoo. Voucher series EWNHM-ANIMAL 6678 – EWNHM-ANIMAL 6679 (total two specimens); Loc: Noewoon-ri, Pyeongchang, Gangwon-do, 7 Apr. 1995; Leg: Seo Suyeon, Yun Seokjun.

***Bufo* sp.**

Two specimens

Voucher series EWNHM-ANIMAL 6625 – EWNHM-ANIMAL 6626 (Juv.; total two specimens); Loc: Sudeoksa, Chungnam, 3 Aug. 2006 – 1 Sep. 2006; Leg: Kim Byungwoo.

**Remarks**: the morphological characteristics to distinguish between *B. gargarizans* and *B. stejnegeri*, such as clearly visible tympanum, were insufficient to identify these specimens at species level.

##### Family Hylidae Rafinesque, 1815

***Dryophytes suweonensis* (Kuramoto, 1980)**

Suweon treefrog; 수원청개구리; one specimen

Original label name: *Hyla suweonensis*

Kuramoto M (1980) Mating calls of treefrogs (genus *Hyla*) in the Far East, with description of a new species from Korea. Copeia 1980: 100–108.

EWNHM-ANIMAL 6377; Loc: Imjingak, Paju, Gyeonggi, 6 Jul. 2011; Leg: no data.

**Remarks**: updated generic assignment according to Duellman et al. (2016).

***Dryophytes japonicus* (Günther, 1859 (1858))**

Japanese treefrog; 청개구리; 26 specimens

Original label name: *Hyla japonica* or *Hyla arborea japonica*

Günther ACLG (1859 “1858”) Catalogue of the BatrachiaSalientia in the Collection of the British Museum. Taylor and Francis, London, United Kingdom, xvi + 160 pp.

Voucher series EWNHM-ANIMAL 6374 – EWNHM-ANIMAL 6375 (total two specimens); Loc: Dobongsan, 16 May. 1959; Leg: Kim and Lee. EWNHM-ANIMAL 6376; Loc: Geomundo lighthouse, 16 Jul. 1977; Leg: Song Junim. EWNHM-ANIMAL 6386; Loc: Cheonmasan, Gyeonggi, 9 May. 1980; Leg: Yun Seokjun. Voucher series EWNHM-ANIMAL 6664 – EWNHM-ANIMAL 6665 (total two specimens); Loc: Wolchulsan, Yeongnam, 5 Oct. 2006; Leg: Kim Byungwoo. EWNHM-ANIMAL 6676; Loc: Imjingak, Paju, Gyeonggi, 6 Jul. 2011; Leg: no data. EWNHM-ANIMAL 6677; Loc: Maesol Forest, Andong, Gyungbuk, 13 Jul. 2013; Leg: Yun Seokjun. EWNHM-ANIMAL 6680; Loc: Noewoon-ri, Pyeongchang, Gangwon-do, 7 Apr. 1995; Leg: Seo Suyeon, Yun Seokjun. EWNHM-ANIMAL 6683; Loc: Gwangneung, 11 May. 1957; Leg: Bae Yeongsoon, Moon Yeongja. EWNHM-ANIMAL 6684; Loc: no data; Leg: no data. EWNHM-ANIMAL 6685; Loc: no data; Leg: no data. Voucher series EWNHM-ANIMAL 6686 – EWNHM-ANIMAL 6687 (total two specimens); Loc: Dobongsan, 17 Jul. 1959; Leg: Kim Yeonghee. EWNHM-ANIMAL 6692; Loc: Godongsan, Gyeonggi-do, 2 May. 1981; Leg: Yun Seokjun. EWNHM-ANIMAL 6693; Loc: Yangsuri, Gyeonggi, 13 Oct. 1978; Leg: Noh Bunjo, Yun Seokjun. EWNHM-ANIMAL 6694; Loc: Dukcheon, Geomundo, 15 Jul. 1977; Leg: Yun Seokjun.

**Remarks**: updated generic assignment as explained above for *D. suweonensis*.

##### Family Microhylidae Günther, 1858 (1843)

***Kaloula borealis* (Barbour, 1908)**

Boreal digging frog; 맹꽁이; 21 specimens

Barbour T (1908) Some new reptiles and amphibians. Bulletin of the Museum of Comparative Zoology 51: 315–325.

Voucher series EWNHM-ANIMAL 5317 – EWNHM-ANIMAL 5322 (total six specimens); Loc: no data; Leg: no data. Voucher series EWNHM-ANIMAL 5352 – EWNHM-ANIMAL 5363 (total 12 specimens); Loc: Gwangju, Gyeonggi-do, 14 Jul. 1982; Leg: Ko Soon Book. EWNHM-ANIMAL 6381; Loc: Gayang Apt., Seoul, 12 Jul. 1993; Leg: Yun Seokjun. EWNHM-ANIMAL 6401; Loc: Nanjido, Seoul, 24 Jul. 2000; Leg: no data. EWNHM-ANIMAL 6723; Loc: no data; Leg: no data.

##### Family Ranidae Batsch, 1796

***Glandirana emeljanovi* (Nikolsky, 1913)**

Imienpo Station frog; 옴개구리; 227 specimens

Original label name: *Rana rugosa*

Nikolskii AM (1913) *Rana emeljanovi* sp. n.. Annuaire du Musée Zoologique de l’Academie Impériale des Sciences de St. Pétersbourg 18: 148–150.

EWNHM-ANIMAL 5283; Loc: Gwangneung, 11 May. 1958; Leg: no data. EWNHM-ANIMAL 5287; Loc: Gwangneung, 11 May. 1957; Leg: no data. EWNHM-ANIMAL 5295; Loc: no data; Leg: no data. Voucher series EWNHM-ANIMAL 5296 – EWNHM-ANIMAL 5323 (total 28 specimens); Loc: Yongwha-ri, Cheorwon-gun, Kangwon-do, 25 Aug. 1977; Leg: Yun Seokjun. EWNHM-ANIMAL 5333; Loc: Gwangneung, 11 May. 1957; Leg: no data. EWNHM-ANIMAL 5334; Loc: Sanghwanam, Sokrisan, 17 Jul. 1961; Leg: no data. Voucher series EWNHM-ANIMAL 5335 – EWNHM-ANIMAL 5340 (total six specimens); Loc: Dobongsan, 16 May. 1959; Leg: no data. Voucher series EWNHM-ANIMAL 5341 – EWNHM-ANIMAL 5347 (total seven specimens); Loc: Gwangneung, 30 Apr. 1978; Leg: Department of Biology. Voucher series EWNHM-ANIMAL 5515 – EWNHM-ANIMAL 5560 (total 46 specimens); Loc: Gyeryongsan, 27 Oct. 1969; Leg: Kim Hungyu. Voucher series EWNHM-ANIMAL 5927 – EWNHM-ANIMAL 5999 (total 73 specimens); Loc: Soyosan, Dongducheon, 19 May. 1967; Leg: Kim Hungyu. Voucher series EWNHM-ANIMAL 6000 – EWNHM-ANIMAL 6059 (total 60 specimens); Loc: Myeongjisan, Gapyeong, 11 May. 1969; Leg: Kim Hungyu. Voucher series EWNHM-ANIMAL 6145 – EWNHM-ANIMAL 6146 (total two specimens); Loc: Hwangyongdong 25-1, Gyeongju, 26 Apr. 2007; Leg: Yun Seokjun. Voucher series EWNHM-ANIMAL 6300 – EWNHM-ANIMAL 6301 (total two specimens); Loc: Songchu, 30 Sep. 1972; Leg: Yeom Yeonghwa. Voucher series EWNHM-ANIMAL 6302 – EWNHM-ANIMAL 6304 (total three specimens); Loc: Gwangneung, 11 May. 1958; Leg: Kang Yeongsaeng. EWNHM-ANIMAL 6368; Loc: Songchu Valley, 30 Sep. 1972; Leg: Park Myeongju (Dept. of Chemistry Education). Voucher series EWNHM-ANIMAL 6411 – EWNHM-ANIMAL 6427 (total 17 specimens); Loc: Gwangneung, 23 May. 1976; Leg: Dept. of Biology. EWNHM-ANIMAL 6632; Loc: Sinwondong, Goyang-si, Gyeonggi-do, 29 Sep.2000; Leg: Yun Seokjun.

**Remarks**: the labels were updated according to generic assignment by Fei et al. (1990) and species name follows the original description by Nikolskii (1913 as *Rana emeljanovi*).

***Lithobates catesbeianus* (Shaw, 1802)**

American bullfrog; 황소개구리; nine specimens

Original label name: *Rana catesbeiana*

Shaw G (1802) General zoology or systematic natural history. Volume III, Part 1. Amphibia. Thomas Davison, London, United Kingdom, 312 pp.

Voucher series EWNHM-ANIMAL 5372 – EWNHM-ANIMAL 5379 (total eight specimens); Loc: no data; Leg: no data. EWNHM-ANIMAL 6380; Loc: Gimpo, 4 Jul. 1999; Leg: Song Junim.

**Remarks**: the labels were updated following the original generic assignment *Lithobates* by Fitzinger (1843), resurrected by Frost et al. (2006).

***Pelophylax nigromaculatus* (Hallowell, 1861(1860))**

Black-spotted pond frog; 참개구리; 188 specimens

Original label name: *Rana nigromaculata*

Hallowell E (1861 “1860”) Report upon the Reptilia of the North Pacific Exploring Expedition, under command of Capt. John Rogers, U.S. N. Proceedings of the Academy of Natural Sciences of Philadelphia 12: 480–510.

Voucher series EWNHM-ANIMAL 5761 – EWNHM-ANIMAL 5795 (total 35 specimens); Loc: Cheonmasan, Gyeonggi, 12 May. 1968; Leg: Kim Hungyu. Voucher series EWNHM-ANIMAL 5827 – EWNHM-ANIMAL 5926 (total 100 specimens); Loc: Cheonmasan, 17 May. 1967; Leg: Kim Hungyu. Voucher series EWNHM-ANIMAL 6336 – EWNHM-ANIMAL 6337 (total two specimens); Loc: Gwangneung, 10 May. 1958; Leg: no data. EWNHM-ANIMAL 6338; Loc: Jeotgae, Anmyeondo, 26 Jul. 1956; Leg: Kim Hoonsoo. Voucher series EWNHM-ANIMAL 6339 – EWNHM-ANIMAL 6348 (total ten specimens); Loc: Dobongsan, 16 May. 1959; Leg: Kim and Lee. EWNHM-ANIMAL 6353; Loc: Baekryeong-do, 27 May, 1958; Leg: Kim Hoonsoo. EWNHM-ANIMAL 6354; Loc: Sanghwanam, Sokrisan, 17 Sep. 1961; Leg: no data. EWNHM-ANIMAL 6379; Loc: Gwangneung, 11 May. 1957; Leg: Ko Wha Soon. EWNHM-ANIMAL 6382; Loc: Taehadong (Ulleungdo), 11 Aug. 1958; Leg: Noh Bunjo. EWNHM-ANIMAL 6383; Loc: Gwangneung, 11 May. 1957; Leg: Ko Wha Soon (Ko Who Soon). Voucher series EWNHM-ANIMAL 6402 – EWNHM-ANIMAL 6409 (total eight specimens); Loc: no location data (label degraded), 26 Jul. 1971; Leg: no data (label degraded). EWNHM-ANIMAL 6410; Loc: Gwangneung, 23 May. 1976; Leg: Dept. of Biology. EWNHM-ANIMAL 6622; Loc: no data; Leg: no data. EWNHM-ANIMAL 6623; Loc: no data; Leg: no data. EWNHM-ANIMAL 6624; Loc: no data; Leg: no data. Voucher series EWNHM-ANIMAL 6637 – EWNHM-ANIMAL 6656 (total 20 specimens); Loc: Soyosan, Gyeonggi-do, 19 May. 1967; Leg: Kim Hungyu. Voucher series EWNHM-ANIMAL 6688 – EWNHM-ANIMAL 6690 (total three specimens); Loc: Hongcheon, Gangwon-do, 2 Aug. 1999; Leg: Jeong Yuhyeon.

**Remarks**: the labels were updated following the original generic assignment by Fitzinger (1843), considered as distinct genus by Fei et al. (1990) and supported by Frost et al. (2006).

***Pelophylax chosenicus* (Okada, 1931)**

Golden-spotted pond frog; 금개구리; 31 specimens

Original label name: *Rana plancyi*

Okada Y (1931) The tailless batrachians of the Japanese Empire. Imperial Agricultural Experiment Station, Tokyo, 215 pp.

Voucher series EWNHM-ANIMAL 5380 – EWNHM-ANIMAL 5395 (total 16 specimens); Loc: Ganghwa-do, 2 Jul. 1972; Leg: Kim Hungyu. Voucher series EWNHM-ANIMAL 5396 – EWNHM-ANIMAL 5409 (total 14 specimens); Loc: Buyong-ri, Yangsu-myeon, Yangpyeong, Gyeonggi-do, 8 Jun. 1970; Leg: Lee Yonghee. EWNHM-ANIMAL 6696; Loc: Gwangneungnae, 22 May. 1971; Leg: Kim Hungyu.

**Remarks**: updated generic assignment as explained above for *P. nigromaculatus*.. The species name follows the original description by Okada (1931; as *Rana nigromaculata chosenica*) under new combination (Frost et al. 2006).

***Rana coreana* Okada, 1928**

Korean brown frog; 한국산개구리; 147 specimens

Original label: *Rana amurensis* or *Rana amurensis coreana*

Okada Y (1928) Frogs in Korea. Chosen Natural History Society Journal 6: 15–46.

Voucher series EWNHM-ANIMAL 6147 – EWNHM-ANIMAL 6196 (total 50 specimens); Loc: Gwangneung, 27 Jun. 1973; Leg: Kim Hungyu. Voucher series EWNHM-ANIMAL 6197 – EWNHM-ANIMAL 6246 (total 50 specimens); Loc: Gwangneung, 12 May. 1971; Leg: Kim Hungyu. Voucher series EWNHM-ANIMAL 6247 – EWNHM-ANIMAL 6285 (total 39 specimens); Loc: Pyeongnae, Gyeonggi, 12 Jun. 1959; Leg: Jang Hanwi. Voucher series EWNHM-ANIMAL 6286 – EWNHM-ANIMAL 6289 (total four specimens); Loc: Pyeongnae, Gyeonggi, 2 Jun. 1959; Leg: Jang Hanwi. Voucher series EWNHM-ANIMAL 6359 – EWNHM 6361 (total three specimens); Loc: Gwangju, Gyeonggi-do, 12 Nov. 2000; Leg: Yun Seokjun. EWNHM-ANIMAL 6390; Loc: Dobongsan, 17 Jul. 1959; Leg: Kim Yeonghee.

**Remarks**: *Rana coreana* was demonstrated to be distinct from *R. amurensis* by Song et al. (2006), and the labels were updated accordingly.

***Rana huanrenensis* Fei, Ye & Huang, 1990**

Huanren frog; 계곡산개구리; 13 specimens

Original label name: *Rana temporaria ornativentris*

Fei L, Ye C, Huang Y (1990) Key to Chinese amphibians. Publishing House for Scientific and Technological Literature, Chongqing, China, 364 pp.

Voucher series EWNHM-ANIMAL 6323 – EWNHM-ANIMAL 6327 (total five specimens); Loc: Sanghwanam, Sokrisan, 15 Jul. 1961; Leg: no data. Voucher series EWNHM-ANIMAL 6618 – EWNHM-ANIMAL 6621 (total four specimens); Loc: Sinwondong, Goyang-si, Gyeonggi-do, 29 Sep. 2000; Leg: Yun Seokjun. EWNHM-ANIMAL 6633; Loc: Jugeumsan, Namyangju, Gyeonggi-do, 17 Sep. 2004; Leg: Yun Seokjun. EWNHM-ANIMAL 6662; Loc: Sinwondong, Dukyang-gu, Goyang-si, Gyeonggi-do, 17 Aug. 2008 ; Leg: Yun Seokjun. Voucher series EWNHM-ANIMAL 6681 – EWNHM-ANIMAL 6682 (total two specimens); Loc: Noewoon-ri, Pyeongchang, Gangwon-do, 7 Apr. 1995; Leg: Seo Suyeon, Yun Seokjun.

**Remarks**: the labels were updated to reflect the change of species status suggested by Fei et al. (1990). The presence of this species in the Republic of Korea was confirmed by Yang et al. (2000).

***Rana uenoi* Matsui, 2014**

Prevernal frog / Ueno’s brown frog; 북방산개구리; 158 specimens

Original label name: *Rana temporaria ornativentris*

Matsui M (2014) Description of a new Brown Frog from Tsushima Island, Japan (Anura: Ranidae: *Rana*). Zoological Science. Tokyo 31: 613–620.

Voucher series EWNHM-ANIMAL 5796 – EWNHM-ANIMAL 5810 (total 15 specimens); Loc: Namyangju, Gyeonggi, 1 Feb. 1970; Leg: Noh Bunjo. Voucher series EWNHM-ANIMAL 6290 – EWNHM-ANIMAL 6299 (total ten specimens); Loc: Sobaeksan, Chungbuk, 1 Oct. 1971; Leg: Kim Hungyu. Voucher series EWNHM-ANIMAL 6305 – EWNHM-ANIMAL 6310 (total six specimens); Loc: Guri, Gyeonggi, 11 May. 1970; Leg: Kim Hunkyu. Voucher series EWNHM-ANIMAL 6311 – EWNHM-ANIMAL 6315 (total five specimens); Loc: Dobongsan, 16 May. 1959; Leg: Kim Okhee. Voucher series EWNHM-ANIMAL 6316 – EWNHM-ANIMAL 6320 (total five specimens); Loc: Sokrisan, Chungbuk, 6 Apr. 1979; Leg: Noh Bunjo, Yun Seokjun. EWNHM-ANIMAL 6321; Loc: Chinbu-ryong (= Jinburyeong), 24 Sep. 1977; Leg: Natural History Museum. EWNHM-ANIMAL 6322; Loc: Songchu Valley, 30 Sep. 1972; Leg: Jeong Hyesook. Voucher series EWNHM-ANIMAL 6328 – EWNHM-ANIMAL 6330 (total three specimens); Loc: Cheoneunsa, Jirisan, 7 May. 1977; Leg: Yun Seokjun. Voucher series EWNHM-ANIMAL 6331 – EWNHM-ANIMAL 6335 (total five specimens); Loc: Jingwansa, 11 May. 1963; Leg: Noh Bunjo. Voucher series EWNHM-ANIMAL 6349 – EWNHM-ANIMAL 6352 (total four specimens); Loc: Sinjang-eup, Gwangju, Gyeonggi, 25 Oct. 1983; Leg: Kim Hoyeong. EWNHM-ANIMAL 6365; Loc: “Under Ewha Bridge”, 11 Mar. 1964; Leg: Jeong Songgeun. Voucher series EWNHM-ANIMAL 6384 – EWNHM-ANIMAL 6385 (total two specimens); Loc: Gwangneung, 11 May. 1957; Leg: Ko Wha Soon (Ko Who Soon). Voucher series EWNHM-ANIMAL 6387 – EWNHM-ANIMAL 6388 (total two specimens); Loc: Bogwangsa, Gyeonggi, 10 May. 1980; Leg: Yun Seokjun. EWNHM-ANIMAL 6389; Loc: Dobongsan, 17 Jul. 1959; Leg: Kim Yeonghee. EWNHM-ANIMAL 6675; Loc: Seogwipo, Jeju, 30 May. 2013; Leg: no data. EWNHM-ANIMAL 6695; Loc: Gwangneungnae, 22 May. 1971; Leg: Kim Hungyu. EWNHM-ANIMAL 6722 (Egg.); Loc: Sokrisan, Chungbuk, 6 Apr. 1979; Leg: Yun Seokjun. EWNHM-ANIMAL 6729 (Egg.); Loc: under Ewha Bridge, 21 Mar. 1964; Leg: “Student”. EWNHM-ANIMAL 6731 (Egg.); Loc: Munsudae, Jirisan, 8 May. 1976; Leg: Yun Seokjun. Voucher series EWNHM-ANIMAL 6732 – 6823 (Td.; total 92 specimens); Loc: Gwangneung, 11 May. 1957; Leg: Bae Yeongsoon, Moon Yeongja.

**Remarks**: the labels were updated to reflect the change of species status of *Rana* from Tsushima Island, Japan, and the Republic of Korea, demonstrated by Matsui (2014).

***Rana* sp.**

Ten specimens

Voucher series EWNHM-ANIMAL 6363 – EWNHM-ANIMAL 6364 (total two specimens); Loc: Gwangneung, 12 May. 1959; Leg: no data. EWNHM-ANIMAL 6663; Loc: Wolchulsan, Yeongnam, 5 Oct. 2006; Leg. Kim Byungwoo. Voucher series EWNHM-ANIMAL 6666 – EWNHM-ANIMAL 6669 (total four specimens); Loc: Sangwonsa, Odaesan, 28 Sep. 2005; Leg; Kim Byungwoo. EWNHM-ANIMAL 6701; Loc: no data; Leg: no data. EWNHM-ANIMAL 6727 (Egg.); Loc: Gwangneung, 11 May. 1957; Leg: Bae Yeongsoon, Moon Yeongja. EWNHM-ANIMAL 6728 (Td.); Loc: Nogodan, Jirisan, 8 May. 1971; Leg: Noh Bunjo.

**Remarks**: all of the bigger *Rana* specimens (now *R. uenoi* and *R. huanrenensis*) deposited in the EWNHM were originally labelled as “*Rana temporaria ornativentris*”. Under the taxonomic framework of *Rana* used here, this name can be traced to either *Rana uenoi* or *Rana huanrenensis*. *Rana uenoi* and *R. huanrenensis* can be distinguished based on several morphometric characteristics (Yang et al. 2000); however, distinguishing between the two species based on this method was not always possible during cataloging procedure. Therefore, it is likely that some of the specimens identified as *R. uenoi* by us are actually *R. huanrenensis*, and vice versa. Moreover, there is a collection of *Rana* froglet specimens that could not be identified and could be a mix of froglets of *R. uenoi*, *R. huanrenensis*, and *R. coreana*. Also, froglets of *Pelophylax nigromaculatus* and *Glandirana emeljanovi* were mixed with these specimens but identified. The specimens of *Rana coreana* have been labelled as *Rana amurensis coreana*.

##### Family Rhacophoridae Hoffman, 1932 (1858)

***Rhacophorus* sp.**

Two specimens

EWNHM-ANIMAL 6697; Loc: no data; Leg: no data. EWNHM-ANIMAL 6698; Loc: no data; Leg: no data.

#### Order Urodela Duméril, 1805

##### Family Hynobiidae Cope, 1859 (1856)

***Hynobius leechii* Boulenger, 1887**

Korean salamander; 도롱뇽; 44 specimens

Boulenger GA (1887) Description of a new tailed batrachian from Corea. Annals and Magazine of Natural History, Series 5, 19: 1–67.

Voucher series EWNHM-ANIMAL 6428 – EWNHM-ANIMAL 6429 (total two specimens); Loc: Cheonmasan, 2 May. 1971; Leg: Noh Bunjo. EWNHM-ANIMAL 6430; Loc: Myeonmokdong, Seoul, 30 Oct. 1976; Leg: Yun Seokjun. EWNHM-ANIMAL 6431; Loc: Sokrisan, Chungbuk, 6 Apr. 1979; Leg: Noh Bunjo, Yun Seokjun. Voucher series EWNHM-ANIMAL 6432 – EWNHM-ANIMAL 6434 (total three specimens); Loc: Dobongsan (behind Bomunsa), 4 Apr. 1965; Leg: Noh Bunjo. EWNHM-ANIMAL 6435; Loc: Nogodan, Jirisan, 5 May. 1977; Leg: Kim Juwan, Hahm Taesik. EWNHM-ANIMAL 6438; Loc: “Purchased from Sejongro”, 20 Apr. 1957; Leg: Kim Okju. Voucher series EWNHM-ANIMAL 6440 – EWNHM-ANIMAL 6453 (Lar.; total 14 specimens); Loc: Cheonmasan, 2 May. 1971; Leg: Noh Bunjo. EWNHM-ANIMAL 6657; Loc: Woljeongsa, Odaesan, 16 Sep. 2006; Leg: Kim Byungwoo. EWNHM-ANIMAL 6658; Loc: Odaesan, 9 Oct. 2006; Leg: Kim Byungwoo. EWNHM-ANIMAL 6660; Loc: Woljeongsa, 11 Oct. 2005; Leg: no data. EWNHM-ANIMAL 6671; Loc: Noron-ri, Pyeongchang, 7 Apr. 2004; Leg: Yun Seokjun, Seo Suyeon. EWNHM-ANIMAL 6702 (Egg.); Loc: Baekwoondae, 28 Mar. 1957; Leg: Noh Bunjo. Voucher series EWNHM-ANIMAL 6709 – EWNHM-ANIMAL 6721 (total 13 specimens); Loc: no data; Leg: no data. EWNHM-ANIMAL 6725 (Egg.); Loc: no data; Leg: no data. EWNHM-ANIMAL 6726 (Egg.); Loc: Munsudae, Jirisan, 8 May 1976; Leg: Noh Bunjo. EWNHM-ANIMAL 6730 (Egg.); Loc: Nogodan, Jirisan, 8 May. 1976; Leg: Yun Seokjun.

***Onychodactylus koreanus* Min, Poyarkov, & Vieites, 2012**

Korean clawed salamander; 한국꼬리치레도롱뇽; 12 specimens

Original label name: *Onychodactylus fischeri*

Poyarkov NA, Che J, Min M-S, Kuro-o M, Yan F, Li C, Iizuka K, Vieites DR (2012) Review of the systematics, morphology and distribution of Asian Clawed Salamanders, genus *Onychodactylus* (Amphibia, Caudata: Hynobiidae), with the description of four new species. Zootaxa 3465: 1–106.

EWNHM-ANIMAL 6436; Loc: Nogodan, Jirisan, 5 May. 1977; Leg: Kim Juwan, Hahm Taesik. EWNHM-ANIMAL 6437; Loc: Jirisan, 14 Jun. 1976; Leg: Hahm Taesik. Voucher series EWNHM-ANIMAL 6635 – EWNHM-ANIMAL 6636 (total two specimens); Loc: Sangwonsa, Odaesan, 28 Sep. 2005; Leg: Kim Byungwoo. EWNHM-ANIMAL 6659; Loc: Maebong, Odaesan, 8 Aug. 2006; Leg: Kim Byungwoo. EWNHM-ANIMAL 6670 (Lar.); Loc: Gajwa-ri, Mitan-myeon, Pyeongchang, 6 Apr. 2005; Leg: Yun Seokjun, Seo Suyeon. Voucher series EWNHM-ANIMAL 6703 – EWNHM-ANIMAL 6708 (total six specimens); Loc: no data; Leg: no data.

**Remarks**: the labels were updated according to taxonomic revision of the genus by Poyarkov et al. (2012).

##### Family Plethodontidae Gray, 1850

***Karsenia koreana* Min, Yang, Bonnett, Vieites, Brandon, & Wake, 2005**

Korean crevice salamander; 이끼도롱뇽; one specimen

Min MS, Yang SY, Bonett RM, Vieites DR, Brandon RA, Wake DB (2005) Discovery of the first Asian plethodontid salamander. Nature. London 435: 87–90.

EWNHM-ANIMAL 6439; Loc: Daedunsan, Jeonbuk, 3 May. 1978; Leg: Natural History Museum.

**Citation**: Shin et al. (2020c).

**Remarks**: this specimen was originally labelled as “*Hynobius leechii*”. This particular specimen predates the formal description of the species by 27 years (Min et al. 2005; Shin et al. 2020c).

### Class Reptilia Laurenti, 1768

#### Order Squamata Oppel, 1811

##### Family Colubridae Oppel, 1811

***Chrysopelea* sp.**

One specimen

EWNHM-ANIMAL 6569 (Nn.); Loc: no data; Leg: no data.

**Remarks**: this specimen was labelled as “a neonate of *Lycodon rufozanatus*”. However, direct comparisons of head shape, tail length, and body shape with another specimen of neonate *L. rufozonatus* (EWNHM-ANIMAL 6544) suggested that the specimen is not *L. rufozonatus* but instead belongs to the genus *Chrysopelea* (Somaweera et al. 2015).

***Elaphe dione* (Pallas, 1773)**

Steppe ratsnake; 누룩뱀; 12 specimens

Pallas PS (1773) Reise durch verschiedene Provinzen des Russischen Reichs in einem ausfuehrlichen Auszuge: vol. 2. Kaiserl. Akad. Wiss., St. Petersburg, 744 pp.

EWNHM-ANIMAL 6537; Loc: no data; Leg: no data. EWNHM-ANIMAL 6539; Loc: no data; Leg: no data. EWNHM-ANIMAL 6542 (Juv.); Loc: Gajeong-ri, Nam-myeon, Chuncheon-si, Gangwon-do, 21 Jun. 2009; Leg: no data. EWNHM-ANIMAL 6543 (Juv.); Loc: Ewha Womans University campus, 19 Oct. 1990; Leg: Paik Seonghoon, Lee Sanghoon, Choi Dongho, Eom Sangryeol. EWNHM-ANIMAL 6563; Loc: no data; Leg: no data. EWNHM-ANIMAL 6584; Loc: Samak-myeon, Chunseong-gun (= Chuncheon), Gangwon-do, 15 Jul. 1976; Leg: Donation by Prof. Paik Namgeuk. EWNHM-ANIMAL 6585; Loc: Gotan, Chunseong-gun (= Chuncheon), Gangwon-do, 7 Jun. 1978; Leg: Donation by Prof. Paik Namgeuk. EWNHM-ANIMAL 6586; Loc: Yangsuri, Gyeonggi, 6 Sep. 1964; Leg: Jeon Songgeun. EWNHM-ANIMAL 6591; Loc: Yangsuri, Gyeonggi, 6 Sep. 1964; Leg: Jeon Songgeun. EWNHM-ANIMAL 6592; Loc: Gajamul, Susaek, Seoul, 9 Jun. 1957; Leg: Tak Soonja. Voucher series EWNHM-ANIMAL 6601 – EWNHM-ANIMAL 6602 (total two specimens); Loc: Bukhansan, 17 Apr. 1971; Leg: Donation by Prof. Yun Ilbyung.

***Elaphe schrenckii* Strauch, 1873**

Russian ratsnake; 구렁이; five specimens

Strauch A (1873) Die Schlangen des Russischen Reichs, in systematischer und zoogeographischer Beziehung. Mémoires de l’Académie Impériale des Sciences de St. Pétersbourg, 7 Série 21: 1–288.

EWNHM-ANIMAL 6498 (Juv.); Loc: Bukri reservoir, Deokjeokdo, 21 Sep. 2009; Leg: Yun Seokjun, Ryu Jaewon. EWNHM-ANIMAL 6540; Loc: no data; Leg: no data. EWNHM-ANIMAL 6583; Loc: Gotan, Chunseong-gun (= Chuncheon), Gangwon-do, 20 Jul. 1978; Leg: Donation by Prof. Paik Namgeuk. EWNHM-ANIMAL 6590; Loc: Jinburyeong, Gangwon-do, Aug. 1978; Leg: Kim Jeonggyun, Lee Jeongsoon. EWNHM-ANIMAL 6605; Loc: no region data, 21 May. 1955; Leg: Department of Biology.

***Hebius vibakari ruthveni* (van Denburgh, 1923)**

Japanese keelback; 대륙유혈목이; five specimens

Original label name: *Natrix vibakari* or *Natrix vibakari ruthveni*

Van Denburgh J (1923) A new subspecies of watersnake (*Natrix vibakari ruthveni*) from eastern Asia. Proceedings of the California Academy of Sciences 13: 3–4.

EWNHM-ANIMAL 6513; Loc: no data; Leg: no data. EWNHM-ANIMAL 6514; Loc: no data; Leg: no data. EWNHM-ANIMAL 6523; Loc: Seongpanak, Jeju (1000m a.s.l), 19 Jul. 1977; Leg: Kim Sooil. EWNHM-ANIMAL 6571; Loc: Palbongsan, Hongcheon, Gangwon-do, 31 Aug. 1978; Leg: Donation by Prof. Paik Namgeuk. EWNHM-ANIMAL 6581; Loc: Jeju-do, 30 May. 1999; Leg: Yun Seokjun.

**Remark**: updated generic assignment for labels follows Thompson (1913), supported by taxonomic revision of *Amphiesma* (*sensu lato*) by Guo et al. (2014).

***Lycodon rufozonatus* Cantor, 1842**

Red banded snake; 능구렁이; 11 specimens

Original label name: *Dinodon rufozonatum*

Cantor T (1842) General features of Chusan, with remarks on the flora and fauna of that island. Annals and Magazine of Natural History, Series 1, 9: 481–493.

EWNHM-ANIMAL 6499; Loc: Ganghwa, 29 Oct. 1991; Leg: Yun Seokjun. EWNHM-ANIMAL 6517; Loc: no data; Leg: no data. EWNHM-ANIMAL 6532; Loc: no data; Leg: no data. EWNHM-ANIMAL 6533; Loc: no data; Leg: no data. EWNHM-ANIMAL 6534; Loc: no data; Leg: no data. EWNHM-ANIMAL 6535; Loc: no data; Leg: no data. EWNHM-ANIMAL 6544 (Juv.); Loc: Gajeong-ri, Nam-myeon, Chuncheon-si, Gangwon-do, 27 Jun. 2009; Leg: Ryu Jaewon. EWNHM-ANIMAL 6570; Loc: no data; Leg: no data. EWNHM-ANIMAL 6587; Loc: Gangchon, Chunseong-gun (= Chuncheon), Gangwon- do, 30 May. 1977; Leg: Donation by Prof. Paik Namgeuk. EWNHM-ANIMAL 6593; Loc: Sinchon, 15 Jun. 1955; Leg: Kim Hoonsoo. EWNHM-ANIMAL 6594; Loc: Sanghwanam, Sokrisan, 15 Jul. 1961; Leg: Chae Ingi.

**Remark**: updated labels reflect taxonomic revision of the group by Guo et al. (2013), who synonymized *Dinodon* with *Lycodon* based on molecular and morphological results.

***Oocatochus rufodorsatus* (Cantor, 1842**)

Frog-eating ratsnake; 무자치; 11 specimens

Original label name: *Elaphe rufodorsata*

Cantor T (1842) General features of Chusan, with remarks on the flora and fauna of that island. Annals and Magazine of Natural History, Series 1, 9: 481–493.

EWNHM-ANIMAL 6509; Loc: no data; Leg: no data. EWNHM-ANIMAL 6510; Loc: no data; Leg: no data. EWNHM-ANIMAL 6511; Loc: no data; Leg: no data. EWNHM-ANIMAL 6512; Loc: Hwajeon, Gyeonggi-do, 3 Oct. 1980; Leg: Yun Seokjun. EWNHM-ANIMAL 6538; Loc: no data; Leg: no data. EWNHM-ANIMAL 6541 (Nn.); Loc: Gwangneung, 28 Sep. 1958; Leg: no data. EWNHM-ANIMAL 6564; Loc: Bucheon, Gyeonggi-do, 28 Jun. 1974; Leg: Kim Hungyu. EWNHM-ANIMAL 6565; Loc: Sangdo-dong, Seoul, 12 Oct. 1961; Leg: Kim Bo Ok. EWNHM-ANIMAL 6566; Loc: Gwangneung; Leg: Im Okja. EWNHM-ANIMAL 6567; Loc: Gwangneung, 4 Jun. 1960; Leg: Lee Namjun. EWNHM-ANIMAL 6580; Loc: Gwangpan-ri, Chunseong-gun (= Chuncheon), Gangwon-do, 10 Aug. 1978; Leg: Donation by Prof. Paik Namgeuk.

**Remark**: the generic assignment for updated labels follows Helfenberger (2001), which demonstrated distinctiveness of this species in relation to other *Elaphe* species.

***Orientocoluber spinalis* (Peters, 1866)**

Slender racer; 실뱀; four specimens

Original label name: *Zamenis spinalis*

Peters WCH (1866) Mittheilung über neue Amphibien (*Amphibolurus*, *Lygosoma*, *Cyclodus*, *Masticophis*, *Crotaphopeltis*) und Fische (*Diagramma*, *Hapalogenys*) des Kgl. Zoologischen Museums. Monatsberichte der Königlichen Preussische Akademie des Wissenschaften zu Berlin 1866: 86–96.

EWNHM-ANIMAL 6500; Loc: Gwanaksan, Gyeonggi-do, 18 Oct. 1958; Leg: Ko Eungwon. EWNHM-ANIMAL 6507; Loc: Biseondae, Seoraksan, Oct. 1958; Leg: Lee Dalyeong. EWNHM-ANIMAL 6508; Loc: Gotan, Chunseong-gun (= Chuncheon), Gangwon-do, 20 Jul. 1978; Leg: Donation by Prof. Paik Namgeuk. EWNHM-ANIMAL 6568; Loc: outside Jahamun, 14 Jul. 1961; Leg: Yun Jeongin.

**Remark**: the updated labels reflect genus-level revision of this species by Kharin (2011).

***Rhabdophis tigrinus* (Boie, 1826)**

Tiger keelback; 유혈목이; 28 specimens

Original label name: *Natrix tigrina* or *Natrix tigrina lateralis*

Boie H (1826) Merkmale einiger japanischer Lurche. lsis von Oken 18–19: 203–216.

EWNHM-ANIMAL 6524; Loc: no data; Leg: no data. EWNHM-ANIMAL 6525; Loc: no data; Leg: no data. EWNHM-ANIMAL 6526; Loc: no data; Leg: no data. EWNHM-ANIMAL 6527; Loc: no data; Leg: no data. EWNHM-ANIMAL 6528; Loc: no data; Leg: no data. EWNHM-ANIMAL 6529; Loc: no data; Leg: no data. EWNHM-ANIMAL 6530; Loc: no data; Leg: no data. EWNHM-ANIMAL 6531 (Nn.); Loc: no data; Leg: no data. EWNHM-ANIMAL 6553; Loc: Bogwangsa, Gyeonggi-do (unknown collection date and year); Leg: no data. EWNHM-ANIMAL 6557; Loc: no data; Leg: no data. EWNHM-ANIMAL 6572; Loc: no data; Leg: no data. EWNHM-ANIMAL 6573; Loc: no data; Leg: no data. EWNHM-ANIMAL 6588; Loc: Miro, Samcheok, Gangwon-do, 16 Jul. 1978; Leg: Donation by Prof. Paik Namgeuk. EWNHM-ANIMAL 6589; Loc: President’s residence, Ewha Womans Univ. campus, 12 May. 1958; Leg: “Workman”. EWNHM-ANIMAL 6595; Loc: Gwangneung, Gyeonggi-do, 25 Jun. 1980; Leg: Kim Sooil. EWNHM-ANIMAL 6596; Loc: Yangsuri, Gyeonggi, 13 Oct. 1978; Leg: Noh Bunjo, Yun Seokjun. EWNHM-ANIMAL 6597; Loc: Baekryeong-do, 27 May. 1958; Leg: Kim Hoonsoo. EWNHM-ANIMAL 6598; Loc: Mujugucheondong, Jeonbuk, May. 1979; Leg: Yun Seokjun. EWNHM-ANIMAL 6599; Loc: Jinburyeong, Gangwon-do, Jul. 1976; Leg: Kim Jeonggyun, Lee Jeongsoon. EWNHM-ANIMAL 6600; Loc: Ewha Womans University campus, 23 May. 1966; Leg: “Workman”. EWNHM-ANIMAL 6603; Loc: no data; Leg: no data. EWNHM-ANIMAL 6604; Loc: no data; Leg: no data. EWNHM-ANIMAL 6606; Loc: Ewha Womans University campus, 15 Jun. 1955; Leg: Department of Biology. EWNHM-ANIMAL 6607; Loc: Ewha Womans University campus, 21 Jun. 1956; Leg: Hahm Jongseong. Voucher series EWNHM-ANIMAL 6608 – EWNHM-ANIMAL 6609 (Nn.; total two specimens); Loc: Gyeryongsan, Chungnam, hatched in captivity, 27 Jul. 2000; Leg: no data. Voucher series EWNHM-ANIMAL 6615 – EWNHM-ANIMAL 6616 (total two specimens); Loc: dubious location name (potentially Gwanak mountain); Leg: Ko Eungwon.

**Remark**: the generic assignment in the updated labels follows the original description of the genus *Rhabdophis* by Fitzinger (1843), based on taxonomic revision of *Natrix* (*sensu lato*) by Malnate (1960).

***Sibynophis chinensis* (Günther, 1889)**

Chinese many-toothed snake; 비바리뱀; one specimen

Günther ACLG (1889) Third contribution to our knowledge of reptiles and fishes from the Upper Yangtsze-Kiang. Annals and Magazine of Natural History 4: 218–229.

EWNHM-ANIMAL 6497; Loc: Hanlim, Jeju-do, 20 Jun. 1999; Leg: Seo Hyeongseok.

##### Family Viperidae Oppel, 1811

***Gloydius brevicaudus* (Stejneger, 1907)**

Short-tailed mamushi; 살모사; ten specimens

Original label name: *Agkistrodon halys* or *Agkistrodon halys brevicaudus*

Stejneger LH (1907) Herpetology of Japan and adjacent territory. Bulletin of the United States National Museum 58: 1–577.

EWNHM-ANIMAL 6501; Loc: Yeongju, Aug. 1957; Leg: D.J. Kim. EWNHM-ANIMAL 6502; Loc: no data; Leg: no data. EWNHM-ANIMAL 6503; Loc: no data; Leg: no data. EWNHM-ANIMAL 6504; Loc: no data; Leg: Department of Biology. EWNHM-ANIMAL 6505; Loc: Gwangpan-ri, Chunseong-gun (= Chuncheon), Gangwon-do, 20 Jun. 1977; Leg: Donation by Prof. Paik Namgeuk. EWNHM-ANIMAL 6520; Loc: Yangju (unknown collection date and year); Leg: Kim Myeongnim. EWNHM-ANIMAL 6536; Loc: no data; Leg: no data. EWNHM-ANIMAL 6551; Loc: Godaedo, 22 Jul. 1956; Leg: Kim Hoonsoo. EWNHM-ANIMAL 6552; Loc: Yukgokcheon, Euiseong, 20 Aug. 1958; Leg: Oh Soonjo. EWNHM-ANIMAL 6562; Loc: Godaedo, 22 Jul. 1956; Leg: Kim Hoonsoo.

**Remark**: generic assignment in the updated labels is based on Hoge and Romano-Hoge (1981), which was supported by results demonstrated by subsequent authors (Knight et al. 1992; Parkinson, 1999). Meanwhile, species assignment in the updated labels is based on the original description by Stejneger (1907 as *Agkistrodon blomhoffii brevicaudus*) because the name *Agkistrodon* (= *Gloydius*) *halys* used in former labels is only applicable to populations of *Gloydius* in Central Asia (Wüster et al. 1997).

***Gloydius intermedius* (Strauch, 1868)**

Rock mamushi; 까치살모사; four specimens

Original label name: *Agkistrodon saxatilis*

Strauch A (1868) Concerning poisonous snakes distributed in Russia. Trudy Perv. Siezda Russ. Yestestv. Zool., 1: 1–294.

EWNHM-ANIMAL 6454 (Nn.); Loc: no data; Leg: no data. EWNHM-ANIMAL 6506; Loc: Daegwanryeong, 3 Aug. 1977; Leg: Donation by Prof. Paik Namgeuk. Voucher series EWNHM-ANIMAL 6545 – EWNHM-ANIMAL 6546 (Nn.; total two specimens); Loc: Mitan, Pyeongchang, Gangwon-do, 3 Nov. 1999; Leg: Donation by Prof. Paik Namgeuk.

**Citation**: Shin et al. (2020b).

**Remark**: generic assignment in the updated labels as explained above for *G. brevicaudus*. Although some authors consider *G. saxatilis* as valid, here we treat that name as a synonym of *G. intermedius* following Orlov and Barabanov (1999). Voucher EWNHM-ANIMAL 6454 is the first reported specimen of *G. intermedius* with dicephalism (Shin et al. 2020b).

***Gloydius ussuriensis* (Emelianov, 1929)**

Ussuri mamushi; 쇠살모사; 18 specimens

Original label name: *Agkistrodon halys* or *Agkistrodon caliginosus*

Emelianov AA (1929) Snakes of the Far Eastern District. Memoirs of the Vladivostok Section of the Russian State Geographical Society 3: 1–208.

EWNHM-ANIMAL 6515; Loc: no data; Leg: no data. EWNHM-ANIMAL 6516; Loc: no data; Leg: no data. EWNHM-ANIMAL 6518; Loc: Mujugucheondong, Jeonbuk, May. 1979; Leg: Yun Seokjun. EWNHM-ANIMAL 6519; Loc: no data; Leg: no data. EWNHM-ANIMAL 6521; Loc: Palbongsan, Hongcheon, Gangwon-do, 2 Jul. 1978; Leg: Donation by Prof. Paik Namgeuk. EWNHM-ANIMAL 6522; Loc: Balang-ri, Paju, Gyeonggi-do, 5 Oct. 1997; Leg: Yun Seokjun. EWNHM-ANIMAL 6547; Loc: Jinburyeong, Gangwon-do, Jul. 1976; Leg: Kim Seonggyun, Lee Jeongsoon. EWNHM-ANIMAL 6548; Loc: Hwajeon, 19 Oct. 1979; Leg: Kim Sooman. EWNHM-ANIMAL 6549; Loc: Gwangneung, Sep. 1968; Leg: Chae Ingi. EWNHM-ANIMAL 6550; Loc: Gwangneung, Gyeonggi-do, 15 May. 1981; Leg: Yun Seokjun. EWNHM-ANIMAL 6554; Loc: Yeongsil, Hanrasan, Jeju-do, 10 Jul. 1979; Leg: Yun Seokjun. Voucher series EWNHM-ANIMAL 6555 – EWNHM-ANIMAL 6556 (total two specimens); Loc: Mujugucheondong, Jeonbuk, 19 Jul. 1967; Leg: no data. Voucher series EWNHM-ANIMAL 6558 – ANIMAL 6559; Loc: Gupabal, 17 Sep.1962; Leg: Noh Bunjo. EWNHM-ANIMAL 6560; Loc: Gwangneung, 10 May. 1959; Leg: no data. EWNHM-ANIMAL 6561; Loc: Gwangneung, 19 May. 1955; Leg: Tak Soonae. EWNHM-ANIMAL 6574; Loc: Jinburyeong, 12 Aug. 1980; Leg: Yun Seokjun.

**Remark**: generic assignment in the updated labels and invalidity of the name “*Agkistrodon halys*” applied to East Asian *Gloydius* species as explained above for *G. brevicaudus*. The species name used in the updated labels follows the original species description by Emelianov (1929 as *Ancistrodon blomhoffi ussuriensis*).

##### Family Agamidae Gray, 1827

***Draco melanopogon* Boulenger, 1887**

Black-bearded flying dragon; one specimen

Boulenger GA (1887) Catalogue of the lizards in the British Museum (Nat. Hist.) III. Lacertidae, Gerrhosauridae, Scincidae, Anelytropsidae, Dibamidae, Chamaeleontidae. London: 575 pp.

EWNHM-ANIMAL 6578; Loc: no data; Leg: no data.

**Remarks**: a female specimen with pre-existing voucher number R54,0,7.

##### Family Lacertidae Oppel, 1811

***Eremias argus* Peters, 1869**

Mongolian racerunner; 표범장지뱀; 12 specimens

Peters WCH (1869) Eine Mittheilung über neue Gattungen und Arten von Eidechsen. Monatsberichte der Königlichen Preussische Akademie des Wissenschaften zu Berlin. 1869: 57–66.

EWNHM-ANIMAL 6455; Loc: Yeonheedongsan, 6 Jun. (unknown collection year); Leg: Kang Yeongok. EWNHM-ANIMAL 6456; Loc: Gwangneung (sandy plain), 11 May. 1957; Leg: Kim Hoonsoo. EWNHM-ANIMAL 6457; Loc: Juan, 6 Jun. 1957; Leg: Kang Yeongsaeng. EWNHM-ANIMAL 6458; Loc: Sinchon, 28 Apr. 1959; Leg: Noh Bunjo. EWNHM-ANIMAL 6459; Loc: no location data, 25 May 1959; Leg: Kim Gihwan. EWNHM-ANIMAL 6460; Loc: Outside Jahamun, Seoul, 1 Jun. 1956; Leg: Yun Jeongin. EWNHM-ANIMAL 6461; Loc: Deokjeokdo, 8 Jul. 1956; Leg: Kim Hoonsoo. Voucher series EWNHM-ANIMAL 6462 – EWNHM-ANIMAL 6464 (total three specimens); Loc: Sindangdong, Seoul, 5 May. 1957; Leg: Yun Deokhee. EWNHM-ANIMAL 6576; Loc: no data; Leg: Kim Bongjin. EWNHM-ANIMAL 6577; Loc: no data; Leg: no data.

***Takydromus amurensis* Peters, 1881**

Amur grass lizard; 아무르장지뱀; 20 specimens

Peters WCH (1881) Einige herpetologische Mittheilungen. 1. Uebersicht der zu den Familien der Typhlopes und Stenostomi gehörigen Gattungen oder Untergattungen. 2. Ueber eine neue Art von Tachydromus aus dem Amurlande. 3. Ueber die von Herrn Dr. finsch aus Polynesien gesandten Reptilien. Sitzungsberichte der Gesellschaft Naturforschender Freunde zu Berlin 1881: 69–72.

EWNHM-ANIMAL 6465; Loc: Gomgol, Seoraksan, 24 Oct. 1971; Leg: no data. EWNHM-ANIMAL 6466; Loc: Bogwangsa, Gyeonggi-do, 16 Apr. 1983; Leg: Yun Seokjun. EWNHM-ANIMAL 6468; Loc: Myeongseongsan, Pocheon, Gyeonggi-do, 23 Nov. 2001; Leg: Yun Seokjun. EWNHM-ANIMAL 6470; Loc: Yumyeongsan, Gapyeong, Gyeonggi-do, 12 Jul. 1992; Leg: Yun Seokjun. EWNHM-ANIMAL 6471; Loc: Gwangneung, 11 May. 1957; Leg: Kim Hoonsoo. EWNHM-ANIMAL 6472; Loc: Daedunsan, Jeonbuk, 3 May. 1978; Leg; Natural History Museum. Voucher series EWNHM-ANIMAL 6476 – EWNHM-ANIMAL 6477 (total two specimens); Loc: Sokrisan, Chungbuk, 6 Apr. 1979; Leg: Kim Sooil. Voucher series EWNHM-ANIMAL 6478 – EWNHM-ANIMAL 6479 (total two specimens); Loc: Mujugucheondong, 8 May.1979; Leg: Yun Seokjun. Voucher series EWNHM-ANIMAL 6480 – EWNHM-ANIMAL 6481 (total two specimens); Loc: Gwangneung, 7 Apr. 1958; Leg: Kim Hoonsoo. Voucher series EWNHM-ANIMAL 6485 – EWNHM-ANIMAL 6486 (total two specimens); Loc: Gwangneung (unknown collection date and year); Leg: Kim Hoonsoo. Voucher series EWNHM-ANIMAL 6487 – EWNHM 6491 (total five specimens); Loc: Baekdamsa, Seoraksan, 27 May. 1999; Leg: Yun Seokjun. EWNHM-ANIMAL 6575; Loc: Jinburyeong, 12 Aug. 1980; Leg: Yun Seokjun.

***Takydromus wolteri* Fischer, 1885**

Mountain grass lizard; 줄장지뱀; six specimens

Fischer JG (1885) Ichthyologische und herpetologische Bemerkungen. V. Herpetologische Bemerkungen. Jahrbuch der Hamburgischen Wissenschaftlichen Anstalten. 2: 82–121.

EWNHM-ANIMAL 6467; Loc: Cheonmasan, 19 May. 1979; Leg: Yun Seokjun. EWNHM-ANIMAL 6474; Loc: Baekryeong-do (unknown collection date and year); Leg: Kim Hoonsoo. EWNHM-ANIMAL 6475; Loc: Sindang-ri, Chungju, 5 May. 1979; Leg: Yun Seokjun. Voucher series EWNHM-ANIMAL 6482 – EWNHM-ANIMAL 6484 (total three specimens); Loc: Anmyeondo (unknown collection date or year); Leg: Kim Hoonsoo.

***Takydromus* sp.**

Two specimens

EWNHM-ANIMAL 6469; Loc: Sangwonsa, Odaesan, 16 May. 1982; Leg: Yun Seokjun. EWNHM-ANIMAL 6473; Loc: Han River (Hangang), 27 Sep. 1959; Leg: Jeong Yeongae.

**Remarks**: we were unable to identify these two specimens because the number of femoral pores (a characteristic that is clearly different between *T. amurensis* and *T. wolteri*) were not clearly visible. Pholidosis characteristics alone were insufficient to make a clear diagnosis at species level.

##### Family Scincidae Gray, 1825

***Scincella vandenburghi* (Schmidt, 1927)**

Tsushima smooth skink; 도마뱀; two specimens

Schmidt KP (1927) Notes on Chinese reptiles. Bulletin of the American Museum of Natural History 54: 467–551.

EWNHM-ANIMAL 6492; Loc: Jugeumsan, Gyeonggi-do, 17 Sep. 1990; Leg: Yun Seokjun. EWNHM-ANIMAL 6691; Loc: Wolchulsan, Yeongnam, 5 Oct. 2006; Leg: Kim Byungwoo.

***Scincella huanrenensis* Zhao & Huang, 1982**

북도마뱀; four specimens

Zhao E, Huang K (1982) A survey of amphibians and reptiles in Liaoning Province. Acta Herpetologica Sinica 1: 1–23.

Voucher series EWNHM-ANIMAL 6493 – EWNHM-ANIMAL 6496 (total four specimens); Loc: Seorim, Yangyang, Gangwondo, 30 Jun. 2008; Leg: Lee Sangcheol.

***Tiliqua gigas* (Schneider, 1801)**

Blue-tongued skink; one specimen

Schneider JG (1801) Historiae Amphibiorum naturalis et literariae. Fasciculus secundus continens Crocodilos, Scincos, Chamaesauras, Boas. Pseudoboas, Elapes, Angues. Amphisbaenas et Caecilias. Frommanni, Jena, Germany. 374 pp.

EWNHM-ANIMAL 6610; Loc: no data; Leg: no data.

#### Order Testudines Batsch, 1788

##### Family Emydidae Rafinesque, 1815

***Trachemys scripta elegans* (Wied, 1838)**

Red-eared slider; 붉은귀거북; four specimens

Original label name: *Pseudemys scripta*

Wied M (1838) Reise in das innere Nord-America in den Jahren 1832 bis 1834, erster Band. J. Hoelscher, Coblenz, 654 pp.

EWNHM-ANIMAL 6580; Loc: no data; Leg: no data. EWNHM-ANIMAL 6617; Loc: no location data, 10 Mar. 1979; Leg: Shin Sook. EWNHM-ANIMAL 6699; Loc: no data; Leg: no data. EWNHM-ANIMAL 6700; Loc: no data; Leg: no data.

**Remark.** generic assignment in the updated labels is based on the original description of *Trachemys* by Agassiz (1857) following the resurrection of *Trachemys* from synonymy with *Pseudemys* by Iverson (1985). This taxonomic treatment has been supported by subsequent studies (Siedel and Smith 1986; Rhodin et al. 2017).

##### Family Trionychidae Fitzinger, 1826

***Pelodiscus maackii* (Brandt, 1858)**

Northern Chinese softshell turtle; 자라; two specimens

Original label name: *Amyda maackii*

Brandt JF (1858) Observationes quaedam ad generis trionychum species duas novas spectantes. Bulletin de l’Académie Impériale des Sciences de St. Petersbourg, la classe Physico-Mathématique 16: 110–111.

EWNHM-ANIMAL 6611; Loc: Yukgokcheon, Gyeongbuk, 20 Aug. 1959; Leg: Oh Soonjo. EWNHM-ANIMAL 6614; Loc: Yangsuri, 30 Oct.1979; Leg: Yun Seokjun.

**Remark**: generic assignment in the updated labels reflects taxonomy used in Rhodin et al. (2017).

***Pelodiscus sinensis* Wiegmann, 1835**

Chinese softshell turtle; 중국자라; one specimen

Original label name: *Amyda sinensis*

Wiegmann AFA (1834) In: Meyen FJF (Ed.) Beiträge zur zoologie gesammelt auf einer reise um die erde. siebente abhandlung. amphibien. Nova Acta Physico-Medica Academia Caesarea Leopoldino-Carolina (Halle) 17: 185–268.

EWNHM-ANIMAL 6612; Loc: “Pond”, 30 Jul. 1964; Leg: Department of Biology.

**Remark**: generic assignment in the updated labels as explained above for *P. maackii*.

***Pelodiscus* sp.**

One specimen

EWNHM-ANIMAL 6613; Loc: no data; Leg: no data.

**Remark**: morphological characteristics used to identify *Pelodiscus* species (Farkas et al. 2019) were insufficient to correctly identify this specimen at the species level.

## Missing specimens and specimens not documented in the catalogue

Some specimens that were known to have been deposited in the EWNHM are not documented here because they were not found in the museum. For example, we failed to locate the holotype of *Karsenia koreana* (EWNHM 80314; Min et al. 2005) and the holotype, paratype series, and other associated specimens of *Onychodactylus koreanus* (holotype EWNHM 80316; paratype series EWNHM 80315, EWNHM 80317–80318; associated specimens EWNHM 80319–80320, EWNHM 80321–80322, EWNHM 80323–80325, EWNHM 80326; EWNHM 80327–80328; Poyarkov et al. 2012). Our attempts to communicate with species authors did not yield any information regarding the whereabouts of these specimens. At this point, it is uncertain whether these specimens are truly lost or on loan without traceability. However, attempts to designate new type materials should be reserved until the exact whereabouts and status of these specimens are known.

In addition, we were unable to document nine specimens of *Dryophytes japonicus* because the specimen jars could not be opened. This was due to crystalized preservative fluid around the inner wall of the lids. Also, we did not document specimens of *Lithobates catesbeianus* and *Pelophylax nigromaculatus* that had been used for dissection samples. These specimens had been used in the medical school before being deposited in the museum, and are of indeterminate origins with no collection data available.

## Conclusions

**Figure 2.**
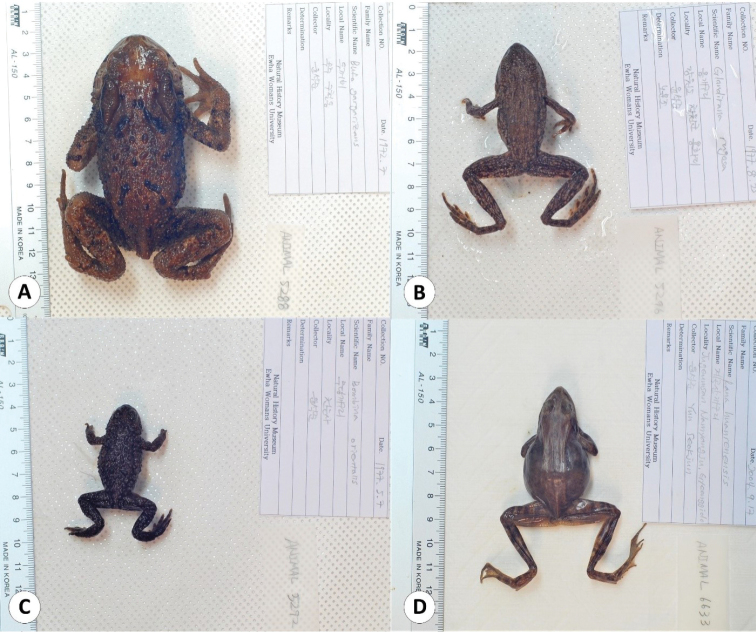
Some of the frog specimens deposited in the collection of the EWNHM**A***Bufo gargarizans* (EWNHM-ANIMAL 5288) **B***Glandirana emeljanovi* (EWNHM-ANIMAL 5296) **C***Bombina orientalis* (EWNHM-ANIMAL 5292) **D***Rana huanrenensis* (EWNHM-ANIMAL 6633).

**Figure 3.**
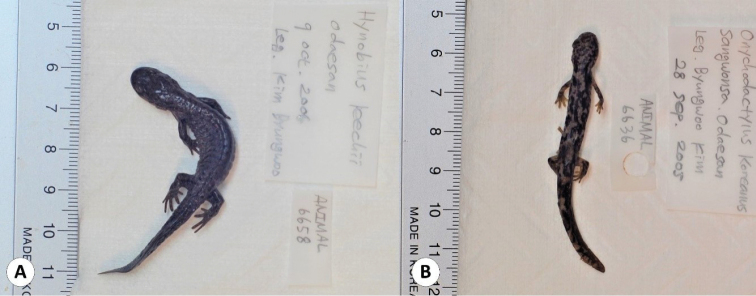
Some of the salamander specimens deposited in the collection of the EWNHM**A***Hynobius leechii* (EWNHM-ANIMAL 6658) **B***Onychodactylus koreanus* (EWNHM-ANIMAL 6636).

**Figure 4.**
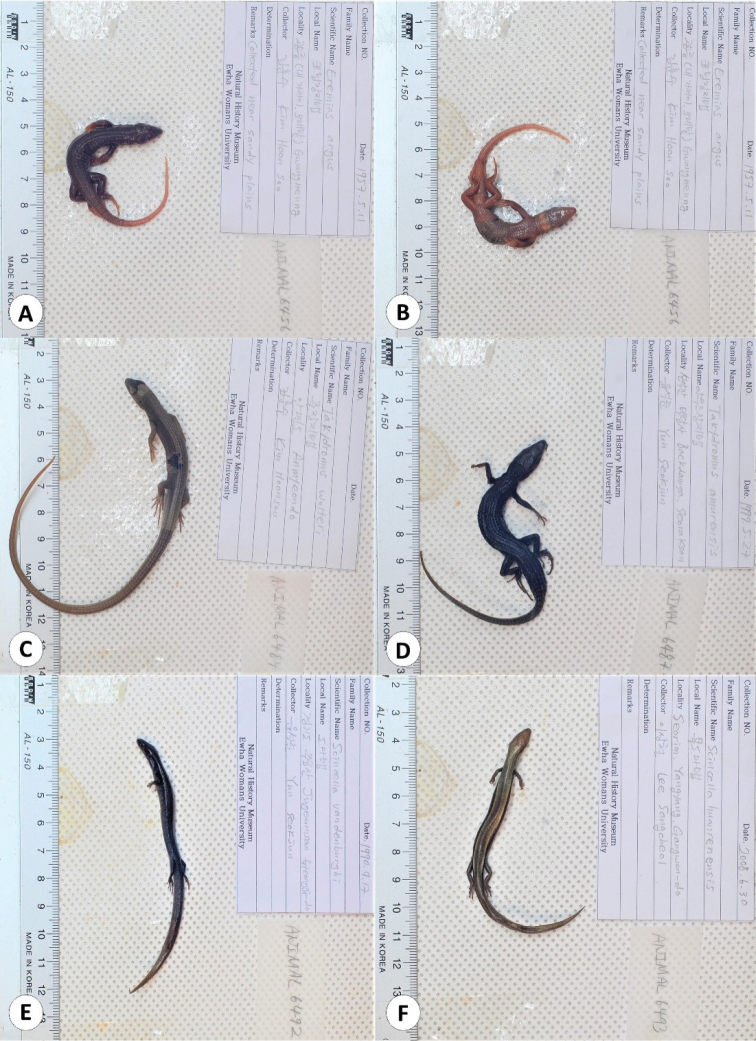
Lizard species of the Republic of Korea represented in the collection of the EWNHM**A***Eremias argus* (EWNHM-ANIMAL 6456; dorsal view) **B***E. argus* (EWNHM-ANIMAL 6456; ventral view) **C***Takydromus wolteri* (EWNHM-ANIMAL 6484) **D***Takydromus amurensis* (EWNHM-ANIMAL 6487) **E***Scincella vandenburghi* (EWNHM-ANIMAL 6492) **F***Scincella huanrenensis* (EWNHM-ANIMAL 6493).

**Figure 5.**
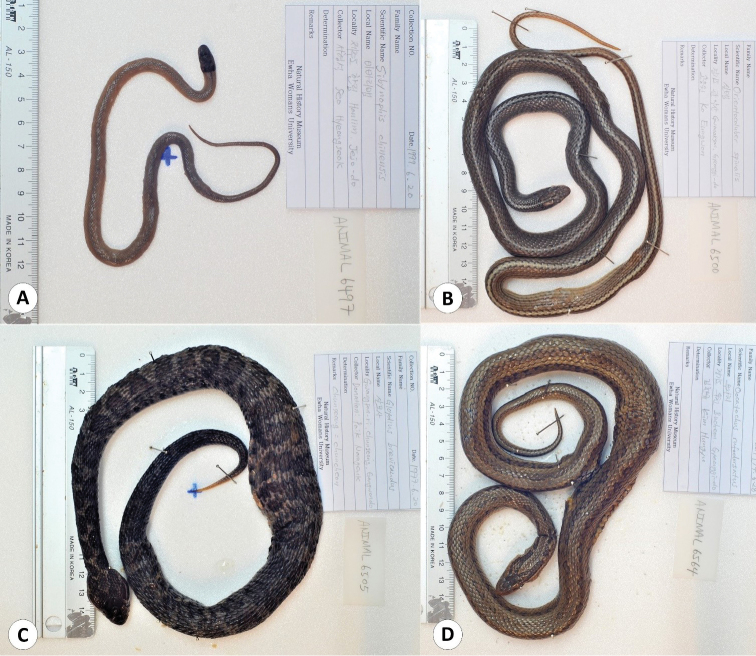
Some of the snake specimens deposited in the collection of the EWNHM**A***Sibynophis chinensis* (EWNHM-ANIMAL 6497) **B***Orientocoluber spinalis* (EWNHM-ANIMAL 6500) **C***Gloydius brevicaudus* (EWNHM-ANIMAL 6505) **D***Oocatochus rufodorsatus* (EWNHM-ANIMAL 6564).

**Figure 6.**
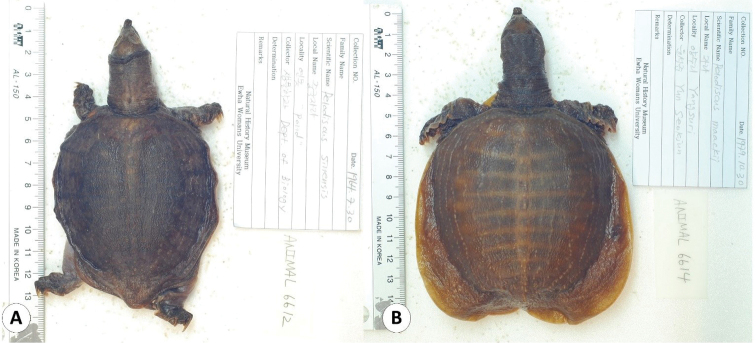
Some of the turtle specimens deposited in the collection of the EWNHM**A***Pelodiscus chinensis* (EWNHM-ANIMAL 6612) **B***Pelodiscus maackii* (EWNHM-ANIMAL 6614).

This catalogue is the first complete inventory of herpetology specimens deposited in the EWNHM. In total, the collection is comprised of 1554 specimens representing all native Korean terrestrial reptiles except one chelonian (*Mauremys reevesii*), all native anuran species, three of six native salamander species, and some exotic and invasive species (Figs 2–6). Some Korean herpetofauna, such as *Hynobius unisacculus* (Min et al. 2016) were not included in the collection because they were recently described and no collection have been made by the institution to acquire vouchers. The specimens of non-native species are most likely from live exhibits of the museum or from laboratory experiments (e.g., specimens of *L. catesbeianus* used in laboratory dissections). In taxonomic diversity, the herpetological collection of EWNHM contain 17 amphibian species across 12 genera and eight families and 22 reptile species across 16 genera and seven families. The specimens were collected between 1951 and 2013. Although the sampling interval is not even, the time span covered by the collection is one of the broadest among collections of Korean herpetofauna. Therefore, the EWNHM collection represents one of the most significant research collections of Korean reptiles and amphibians.

Natural history collections are a valuable resource for a number of reasons and by cataloging the collection at EWNHM, the specimens held there are now accessible to researchers for perpetuity. In recent times, natural history collections have proved valuable resources in order to trace the origins and spread of disease (Ouellet et al. 2005) as well as to reconstruct the genetic diversity in long-extinct populations (Wandeler et al. 2007). With advancing molecular methods, formalin-fixed specimens may soon be able to provide the wealth of knowledge that we can currently extract for those fixed in ethanol. As we shift towards a more holistic approach to conservation, supported by global projects, it is hoped that the above catalogue can aid in herpetofauna conservation for decades to come.
